# Pandemiebedingtes Verkaufsverbot von Feuerwerkskörpern in Deutschland führt zu einer deutlichen Abnahme der Augenverletzungen

**DOI:** 10.1007/s00347-022-01778-1

**Published:** 2022-11-30

**Authors:** Ameli Gabel-Pfisterer, Daniel Böhringer, Hansjürgen Agostini, Feuerwerks-Verletzungen-Studiengruppe, D. Kuerten, M. Fuest, P. Walter, K. Wanjek-Meyer, L Kohen, K. Hartmann, Y. Botros, G. Eberlein, J. Märtz, C. Kojetinski, A. Mueller, J. Hagenbusch, M. Gritzka, C. Dempe, N. Al-Ashi, H. Breuß, J. Kuchenbecker, M. H. Foerster, I. Seibel, A. Böker, D. Greve, M. Lenglinger, B. Müller, S. Schönfeld, I. Seibel, A. Joussen, D. Kolarov, P. Schwarz, C. Puk, A. Berthold, C. Wirbelauer, H. Hofmayer, J. Wachtlin, J. Wachtlin, J. F. Meyer, T. Macher, K. Palka, M. Niemeyer, T. Walla, D. Pham, S. Aisenbrey, P. Rieck, J. Verbeck, A. Tatsiou, A. Walch, R. Burk, G. Schnober, M. Elling, T. Schultz, N. Tsiampalis, J. Rehmann, U. Sliwowska, M. Schojai, K. Schulze, N. Kamguia, C. Wirtz, B. Dick, L. Bourauel, F. M. Schützeichel, D. Völcker, M. Wintergerst, M. Pfau, C. Melzer, D. Hoegen, F. Bosch, J. C. Andresen, T. Krohne, F. G. Holz, U. Fries, M. Koch, A. Kwasnicki, M. Kathke, W. Noske, A. Sturm, E. Chankiewitz, S. Monastoriotis, O. Kemper, T. Hübner, M. Feldmann, J. Morsek, O. Rainer, H. Bartsch, K. Ewald, E. Chankiewitz, S. Brandter, M. U. Cil, T. Siegmund, A. Bohlen, A. Mohr, A. Wienigk, J. Hecker, P. Smetana, O. Furashova, K. Engelmann, M. Shtaya, A-K. Müller, A. Krieb, K-H. Emmerich, L. Grajewski, L. Krause, D. Q. Hoa, S. Yilmaz, A. Jabur, K. Rüdiger, T. Boeker, D. Rashitova, F. Lehmann, H. Sachs, E. Matthee, L. Pillunat, L. Juergens, S. Kaya, R. Guthoff, F. Steindorf, J. Korbmacher, G. Geerling, R. Widder, G. Rössler, A. Iseed, J. Doulgkeridis, J. Erhard, M. Tomalla, B. von Jagow, F Filev, S Schill, T Kotiasvili, A. Flach, C. Zollfrank, A. Lieder, M. Blum, T. Tourtas, H. Knorr, F. Kruse, M. Freimuth, S. Dalbah, E. Sokolenko, P. Rating, T. Kiefer, B. Book, H. Westerkemper, M. Böhm, N. Bornfeld, N. Bechrakis, M. Schultheiss, A. Scheider, K. Pawlowczicz, M. Müller, T. Kohnen, K. Ahdab, T. Eckert, C. Eckardt, M. Wisniewska, A. Just, Y. Laich, J. Stifter, M. Avar, V. Jehle, T. Reinhard, S. Rab, J. Seewald, C. Mais, S. Basiakos, B. Osman, E. Xanthopoulou, B. Friedburg, M. H. Graef, B. Lorenz, U. Just, J. Schrecker, J. Klemming, D. Drüke, L. Bemmer, S. Weiß, P. Take, A. Nguyen-Höhl, C. Oterendorp, N. Feltgen, H. Hoerauf, I. Prusiecki, J. Elle, B. Gundel, M. C. Bender, A. Menges, F. Tost, A. Stahl, R. Wienrich, A. Huth, A. Viestenz, J. Ueberschaar, T. Daehn, U. Brooks, P. Schindler, E. Bigdon, P. Bertram, C. Skevas, R. Kromer, M. Casagrande, C. Grohmann, J. Mehlan, P Schindler, M. Spitzer, M. Schargus, M. Eddy, S. Schumacher, M. Keserü, A. Scheler, B. Stemplewitz, U. Schaudig, J. Herden, M. Haar, B. Tode, B. Junker, W. Abou Mouli, I. Volkmann, C. Framme, A. Scheuerle, M. Auerbach, C. Beisse, K. Rohrschneider, G. Auffahrt, N. Mala, A. Rosenthal, L. Hesse, L. Daas, E. Flockerzie, S. Suffo, B. Seitz, N. Chrisoglou, G. Wietstock, R. Augsten, D. Meller, I. Althauspetervari, O. Rudolph, C. Floeter, A. Beutner, R. Effert, M. Mayer, K. Vanselow, W. Lieb, C. Kandzia, K. Purtskhvanidze, C. Ehlken, J. Roider, A. Hueber, K. Cursiefen, C. Edelmann, N. Schrage, M. Kroeger, N. Viehweg, P. Meier, J. D. Unterlauft, P. Wiedemann, M. Rehak, F. Ziemssen, F. Rommel, S. Sonntag, M. Prasuhn, V. Pawlik, V. Kakkassery, M. Ranjbar, A. Mohi, S. Grisanti, I. Bastron, S. Dindin-Sarac, S. Kaskel-Paul, J. Rawohl, L. Hattenbach, B. Stoffelns, A. Schuster, N. Pfeiffer, V. Besgen, F. Schröder, S. Schulze, N. Weber, W. Sekundo, C. Schuart, G. Renieri, M. Weigel, H. Thieme, F. Hagenau, A. Wolf, E. Vounotrypidis, S. Priglinger, J. Penkava, J. Klein, L. Bechstein, M. Maier, C. Lohmann, C. Haritoglu, F. Alten, N. Eter, C. Brinkmann, F. Alshikh, V. Klishko, U. Holland, A. Medra, A. Weber, H. Höh, A Pielen, B Junker, T. Zschockelt, F. Luciani, J. Schmidbauer, P. Horn, L. Kodomskoi, H. Kuempel, C. Rivera Gomez, K. Plantzas, M. Weiss, K. Hille, G. Esper, K. Mazko, L. Kolbeck, S. Malek, P. Kupper, S. Grafmueller, S. Schrader, R. Darawsha, N. Bellios, V. Wulff, A. Ghaffary, A. Ghoreishi, F. Höhn, A. Napholz, T. Tandogan, L. Schmidt, P. Ilski, C. Trossowski, M Zühlsdorff-Utke, A. Liekfeld, I. Winter, A. Böhm, C. Blecha, T. Barth, H. Helbig, W. Rusch, A. Noerenberg, A. Juenemann, T. A. Fuchsluger, C. Matar, M. Zuche, S. Roehrig, A. Decker, M. Kühn, M. Ladewig, J. Schmidt-Wetter, R. Machulla, A-F. Boateng, M. Dias Blak, S. Krawczyk, K. Lenhard, B. Lackner, F. Gekeler, D. Mamacek, L. Wocker, I Holzschuh, K. T. Boden, P. Szurmann, D. Faul, K. May-Endres, U. Press, J. Luttke, L. Wolfram, F. Reichel, I. Seitz, F. Ziemssen, U. Bartz-Schmidt, A. Speidel, J. Cordes, F. Raber, M. Mikielewicz, J Kammerer, S. Kupferschmid, H. Buchwald, F. Raber, J. Werner, J. Kampmeier, A. Wolf, S. Dithmar, G. Fischer, C. Pruefke, A. Bula, P Krauß, P. Strzalkowski, J. Hillenkamp

**Affiliations:** 1 Augenklinik, Klinikum Ernst-von-Bergmann, Charlottenstr. 72, 14467 Potsdam, Deutschland; 2 Klinik für Augenheilkunde, Medizinische Fakultät, Universitätsklinikum Freiburg, Freiburg im Breisgau, Deutschland

**Keywords:** Augenverletzungen durch Feuerwerk, Verkaufsverbot privates Feuerwerk, Feuerwerks- und Knallkörper, Augentrauma durch Explosion, Augenverletzung und pyrotechnische Artikel, Firework induced eye-injuries, Salesban consumer fireworks, Fireworks and crackers, Eyetrauma and explosion, Eye injuries and pyrotechnics

## Abstract

**Hintergrund und Ziel:**

Die kontinuierliche Erfassung von Augenverletzungen durch Pyrotechnik in den Tagen um Silvester über 6 Jahre ermöglicht uns, Verletzungszahlen, Verletzungsmuster und Unfallhergänge im Jahresvergleich zu untersuchen. Zur Entlastung der Krankenhäuser wurden in Deutschland im Rahmen der COVID-19-Pandemie für die Jahreswechsel 2020/21 und 2021/22 ein Verkaufsverbot für Pyrotechnikartikel und Versammlungsbegrenzungen umgesetzt. Wir untersuchen, welchen Einfluss diese Maßnahmen auf die Anzahl von feuerwerksbedingten Augenverletzungen hatten. Außerdem betrachten wir, ob dies zu einer Zunahme schwerer Verletzungen geführt hat und ob ein Zusammenhang mit einer vermehrten Nutzung selbst gebauter oder in Deutschland nicht zugelassener Pyrotechnik bestehen könnte.

**Methoden:**

Mit unserem Online-Fragenbogen erfassen wir anonymisierte Daten zu Patienten, Unfallhergang und seit 2017/18 auch zur Beschaffung der eingesetzten Pyrotechnikartikel.

**Ergebnisse:**

Unsere Auswertung umfasst Daten von 2151 Betroffenen. Während vor der Pandemie pro Jahr Daten von rund 500 Verletzten eingegeben worden waren, sank diese Zahl auf 79 am Jahreswechsel 2020/21 und 193 2021/22. Der Anteil schwerer, stationär zu versorgender Augenverletzungen lag in den Jahren vor der Pandemie zwischen 21 und 26 %, in den Pandemiejahren 2020 bis 2022 hingegen bei 27 % und 34 %. Gleichzeitig stieg unter dem Verkaufsverbot der Anteil von Pyrotechnikartikeln, die nach Aussagen der Patienten selbst gebaut oder inoffiziell erworben waren, von 3 % auf knapp 10 % an. Bei den absoluten Zahlen jedoch stehen 67 Unfälle mit nicht offiziell erworbener Pyrotechnik 1675 Zwischenfällen mit offiziell erworbenen oder nicht benennbaren Feuerwerkskörpern gegenüber. In etwa der Hälfte der Fälle konnte zum auslösenden Pyrotechnikartikel keine Aussage gemacht werden, was erklärbar ist durch den hohen Anteil (rund 50 %) verletzter Unbeteiligter.

**Schlussfolgerung:**

Die absolute Zahl der Augenverletzungen durch Pyrotechnik sank unter den Pandemiebedingungen von rund 500 auf 79 bzw. 193. Die Nutzung von Feuerwerkskörpern, die als nicht offiziell erworben bezeichnet wurden, war auch unter dem Verkaufsverbot anteilsmäßig gering und spielt im Vergleich zu Verletzungen mit „offiziell erworbener“ Pyrotechnik eine untergeordnete Rolle.

**Zusatzmaterial online:**

Zusätzliche Informationen sind in der Online-Version dieses Artikels (10.1007/s00347-022-01778-1) enthalten.

## Abstract (en)

**Background and aim:**

The continuous recording of eye injuries caused by fireworks around New Year’s Eve over 6 years allows us to investigate injury numbers, injury patterns, and accident histories in a year to year comparison. In order to relieve the burden on hospitals during the COVID-19 pandemic, a ban on the sale of firework articles was implemented in Germany for 2020/2021 and 2021/2022.

We investigate the impact of these measures on the number of fireworks-related eye injuries and analyze whether this led to an increase in serious injuries and an association with increased use of homemade pyrotechnics or pyrotechnics potentially not approved in Germany.

**Methods:**

We used our online questionnaire to collect anonymized data on patients, accident history, and, since 2017/2018 information on the acquisition of firework articles.

**Results:**

Our analysis included data from 2151 affected individuals. While data of about 500 injured were entered per year before the pandemic, this number decreased to 79 in 2020/2021 and 193 in 2021/2022. The proportion of severe eye injuries requiring inpatient care ranged from 21% to 26% in the years before the pandemic but ranged from 27% to 34% in the pandemic years. At the same time, under the sales ban the proportion of fireworks that were homemade or unofficially purchased increased from 3% to nearly 10%. In terms of absolute numbers, however, there were 67 accidents involving unofficially purchased pyrotechnics compared with 1675 incidents involving officially purchased or unidentified fireworks, which can be explained by the high proportion (about 50%) of injured bystanders.

**Conclusion:**

The absolute number of patients with eye injuries caused by pyrotechnics decreased under the pandemic conditions from about 500 to 79 or 193. The use of fireworks designated as unofficially purchased was proportionally low even under the sales ban, and plays a minor role compared to injuries with officially purchased fireworks articles.

## Hintergrund und Ziel

Privates Feuerwerk führt regelmäßig zu Verletzungen vor allem der Augen, des Gesichts und der Hände. Unsere Arbeitsgruppe führt zusammen mit klinikbasierten Notfallversorgern aus ganz Deutschland seit 2016 eine prospektive online-basierte Umfrage durch, die Verletzungen durch Feuerwerk an Augen, Gesicht und Hand erfassen soll. Die 3‑Jahres-Ergebnisse wurden erstmals 2019 veröffentlicht [1].

Die kontinuierliche Erfassung über die vergangenen 6 Jahre hinweg ermöglicht uns, Umfragedaten wie Patientenanzahl, -geschlecht und -alter, Verletzungsmuster und Unfallhergang im Jahresvergleich und auch unter dem Wechsel zu den pandemiebedingten gesetzlichen Regelungen zu untersuchen. Es bietet sich damit eine gute Möglichkeit, den prophylaktischen Effekt dieser Maßnahmen zu beurteilen. Zur Entlastung der Krankenhäuser in der SARS-CoV-19-Pandemie beschloss die Bundesregierung für die Jahreswechsel 2020/21 und 2021/22 jeweils ein Maßnahmenpaket. Es beinhaltete ein Verkaufsverbot für Feuerwerk der Kategorie 2 und Versammlungsverbote von mehr als 5 Personen aus 2 Haushalten [2].

Vor Einführung der pandemiebedingten Regelungen ist verschiedentlich angeführt worden, dass ein Verkaufsverbot offizieller pyrotechnischer Artikel zu einer Verschiebung der „Bölleraktivität“ auf selbst gebaute oder in Deutschland nicht zugelassenen Produkten führen würde und damit ein höheres Verletzungsrisiko mit sich bringe. Nachdem nun Daten zu 2 Jahreswechseln unter Pandemiebedingungen vorliegen, wird dieser Zusammenhang im Vergleich mit den Daten aus den 4 vorangehenden Jahren untersucht.

## Material und Methoden

Unsere prospektive Studie basierte wie in den Vorjahren auf der internetgestützten, standardisierten Umfrage an allen deutschen notdienstleistenden Augenkliniken.

Alle Klinikleitungen wurden gemäß der aktualisierten Liste der Deutschen Ophthalmologischen Gesellschaft (DOG) kontaktiert und mit ihren ärztlichen Mitarbeitenden eingeladen, an der Studie teilzunehmen, welche einen Zeitraum von 5 Tagen um Silvester umfasst.

Die Erfassung erfolgte durch die Notdienst leistenden Augenärztinnen und Augenärzte. Es wurden anonymisiert Daten zu Alter, Unfallhergang, Art der Feuerwerkskörper und augenärztlichem Befund abgefragt (Online zu diesem Beitrag ist der Fragebogen zu finden). Die Auswertung erfolgte in dieser Betrachtung wegen der im Vergleich zu den Vorjahren niedrigeren Gesamtzahl überwiegend in der Gesamtgruppe aller Altersstufen. Da die Antworten optional waren, bezieht sich die Auswertung auf die jeweils rückläufigen Daten einer Frage. Die statistische Auswertung erfolgte deskriptiv für kategoriale Variablen, die prozentualen Anteile wurden dargestellt. Für kontinuierliche Variablen wurden Mittelwerte und Standardabweichungen berechnet. Wir verwendeten den Chi-Quadrat-Test zur Prüfung des Zusammenhangs zwischen 2 Faktoren. *p*-Werte unter 0,05 wurden als statistisch signifikant betrachtet. Eine Korrektur für multiples Testen erfolgte nicht. Für alle Analysen verwendeten wir die Programmiersprache „R“.

Zum Jahreswechsel 2017/18 wurde eine Frage zur Beschaffung der auslösenden Feuerwerkskartikel in den Fragebogen aufgenommen, um auszuwerten, ob offiziell erworbene, nicht offiziell (z. B. im Ausland oder online) erworbene oder selbst gebaute Produkte verwendet worden waren.

Eine Erfassung des Wetters, das Einfluss auf die Anzahl der Verletzungen haben kann, erfolgt hier nicht. Bei der deutschlandweiten Erfassung gehen wir von relativ geringem Einfluss durch „Mittelung“ aus.

Eine Überprüfung der Daten ist bei anonymisierter Dateneingabe nicht möglich. Einige Daten stellen sich jedoch über alle Jahre stabil dar, was für Robustheit spricht. In Zweifelsfällen der Plausibilität, z. B. fragliche Mehrfacherfassung, wurde mit den Erstuntersuchenden Rücksprache gehalten.

## Ergebnisse

### Beteiligung der Kliniken und Gesamtzahl der Verletzten

Insgesamt konnten von Jahr zu Jahr mehr Kliniken zur Beteiligung an der Studie gewonnen werden (Tab. 1). Am Jahreswechsel 2020/21 trugen 75 Kliniken zur Umfrage bei und übermittelten Daten zu insgesamt 79 Verletzten. Im Folgejahr übermittelten 77 Kliniken Daten zu 193 Betroffenen. Insgesamt überblicken wir damit Daten von 2151 Patienten.

**Table 1:** 

Jahreswechsel	Meldende Zentren	Gemeldete Fälle	Relative Fallzahl
2016/17	41	350	8,5
2017/18	49	518	10,6
2018/19	52	488	9,4
2019/20	59	523	8,7
2020/21^a^	75	79	1,1
2021/22^a^	77	193	2,5

^a^ Jahreswechsel mit pandemiebedingtem Verbot von Feuerwerk

In den Jahren vor der Pandemie dokumentierten wir nach dem ersten Jahr jährlich rund 500 Patienten mit Augenverletzungen durch Feuerwerkskörper.

Damit zeigte sich in den Jahren unter Pandemiebedingungen ein Rückgang der mittleren Patientenanzahl pro Klinik von 9,3 auf 1,8 und 2,5.

Insgesamt haben zuletzt alle deutschen Universitäts-Augenkliniken und 39 nicht-universitäre Augenkliniken teilgenommen. Damit erreicht diese Umfrage eine Rücklaufquote von 86 % aller deutschen Augenkliniken, die sich am augenärztlichen Notdienst beteiligten.

Als Hotspot stellte sich auch in den Pandemiejahren Berlin dar, gefolgt von Hamburg und München.

### Geschlecht und Altersstruktur

Der überwiegende Anteil der Verletzungen durch Feuerwerk betrifft männliche Patienten. Ihr Anteil betrug am Jahreswechsel 2020/21 65 %, im folgenden Jahr 78 %. In den Vorjahren waren vergleichbar 75 % der Patienten männlich.

Der Anteil der Minderjährigen sank im ersten Pandemiejahr auf ein Viertel aller Betroffenen. Im zweiten Pandemiejahr waren jedoch ähnlich wie in den Jahren vor der Pandemie 35 % der Patienten minderjährig.

**Figure 1:**
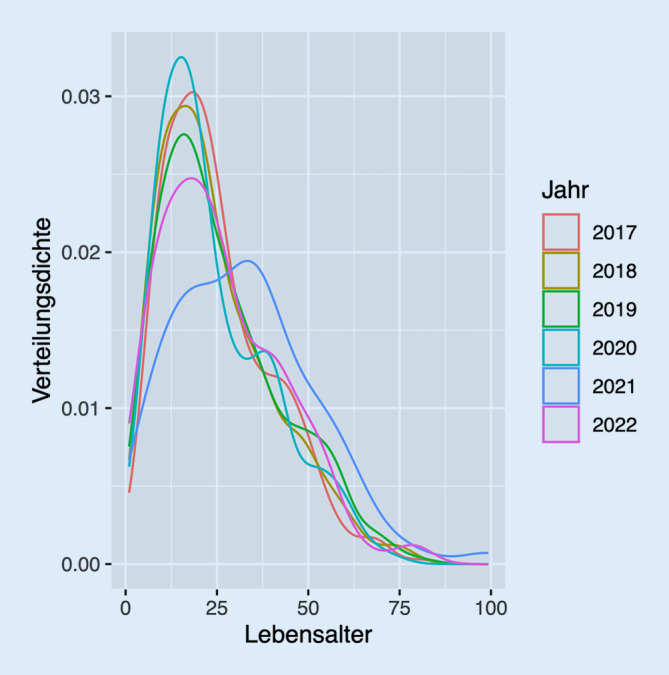

So zeigte auch die Altersverteilung am Jahreswechsel 20/21 einen Gipfel um das 30. Lebensjahr, während er im Folgejahr wie in den Vor-Pandemie-Jahren im jugendlichen Alter lag (Abb. 1).

### Tendenz: mehr stationäre Behandlungen im zweiten Pandemiejahr

**Figure 2:**
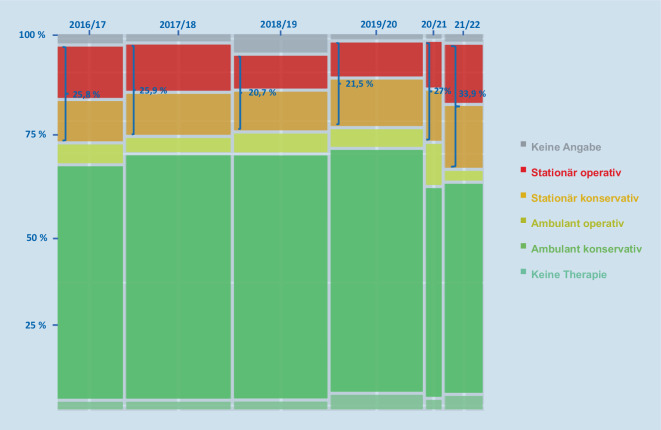

Von den 78 Verletzten, zu denen am Jahreswechsel 20/21 eine Angabe zur erforderlichen Therapie vorlag, mussten 27 % stationär behandelt werden (15 von 59 Erwachsenen und 6 von 19 Minderjährigen). Am Jahreswechsel 21/22 waren 34 % aller Patienten stationär zu behandeln (49 von 122 Erwachsenen und 15 von 67 Minderjährigen). Der Anteil der Schwerverletzten lag damit am letzten Jahreswechsel etwas höher als in den Jahren vor der Pandemie, als er zwischen 21 und 27 % (Mittelwert 23,5 %) betrug (Abb. 2).

### Begleitverletzungen

**Figure 3:**
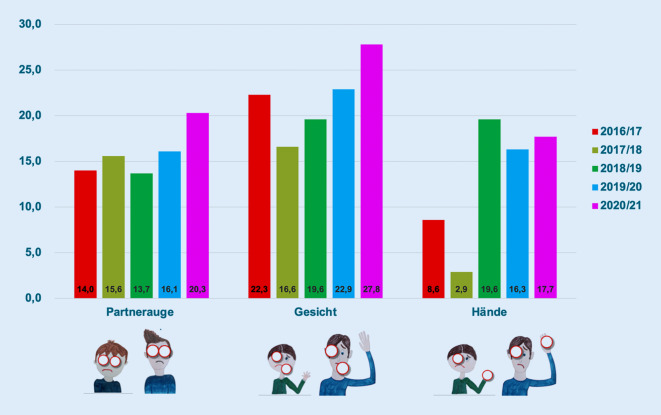

Einen weiteren Hinweis auf den Schweregrad der Verletzungen könnte der Anteil der Betroffenen mit Begleitverletzung geben:

In den Pandemiejahren 2020 bis 2022 trugen von den 272 Patienten 76 (28 %) eine beidseitige Verletzung, 85 (31 %) eine zusätzliche Gesichtsverletzung und 38 Patienten (14 %) eine begleitende Handverletzung davon. In den Jahren vor der Pandemie waren es von insgesamt 1879 Patienten 281 mit beidseitigen Verletzungen (15 %), 380 mit Beteiligung des Gesichts (20 %) und 226 (12 %) mit zusätzlicher Verletzung mindestens einer Hand (Abb. 3).

Insgesamt scheint bei niedrigerer Gesamtzahl der Unfälle der Schweregrad der Verletzungen tendenziell etwas höher zu sein als in den Jahren vor der Pandemie.

### Quelle der ursächlichen Pyrotechnik

**Figure 4:**
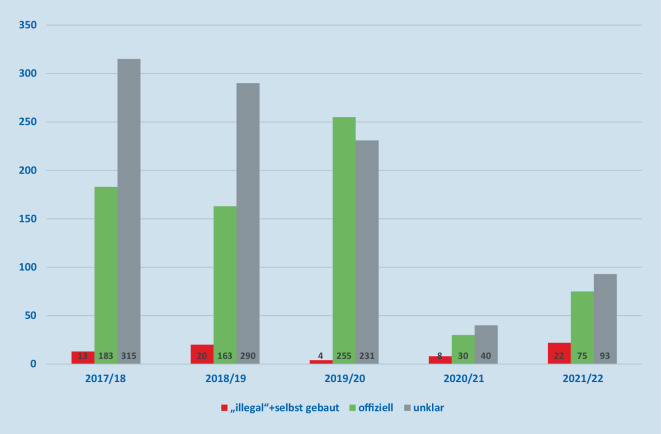

Wir werteten die Antworten auf die Frage nach der Quelle der ursächlichen pyrotechnischen Artikel aus. Aus nicht offizieller Quelle wurde Pyrotechnik eingeordnet, wenn sie im Ausland oder aus dem Internet erworben oder selbst gebaut war. Während der Jahre 2020 bis 2022, in denen ein Verkaufsverbot für Feuerwerksartikel galt, kam bei 30 von 269 (11 %) Unfällen der ursächliche pyrotechnische Artikel aus nicht offizieller Quelle, während das in den Jahren 2017 bis 2020 nur bei 37 von 1528 (2 %) Unfällen so angegeben worden war (Abb. 4). Dieser Unterschied im Anteil von Verletzungen durch offizielles oder nicht offizielles Feuerwerk ist für alle Pandemiejahre im Vergleich zu den Nicht-Pandemiejahren statistisch signifikant (Chi-Square, *p* < 0,0001) (Tab. 2).

**Table 2:** 

	„Offiziell“ erworbene Feuerwerksartikel	„Inoffiziell“ erworbene Feuerwerksartikel	Gesamt
*Vor der Pandemie*	1491	37	1528
*Während der Pandemie*	239	30	269
*Gesamt*	1730	67	1797

*p* < 0,001 (Chi-Quadrat-Test)

**Figure 5:**
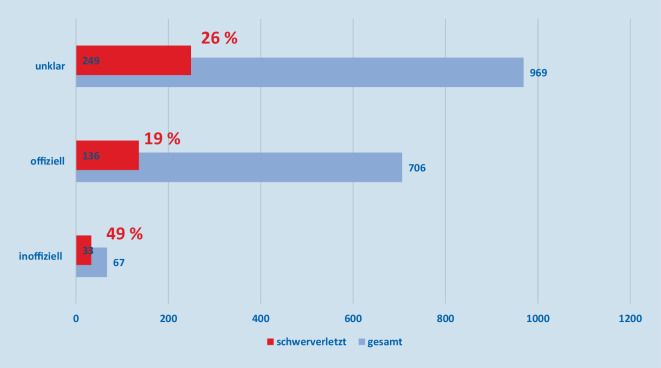

**Figure 6:**
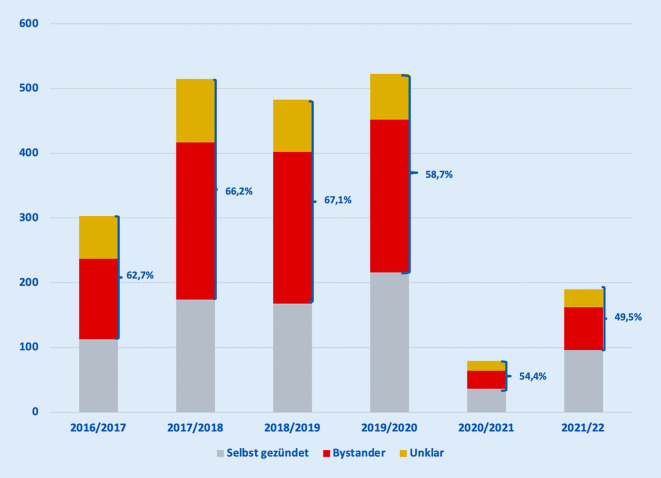

Wir wollten beschreiben, wie viele der schweren Verletzungen auf nichtoffizielle und offizielle Feuerwerkskörper zurückzuführen sind, und analysieren daher die Daten über alle Jahre hinweg gesammelt: Seit 2017 waren Verletzungen durch aus nicht offiziellen Quellen beschaffte Feuerwerksartikel in etwa der Hälfte (49 %) der Fälle so gravierend, dass eine stationäre Behandlung notwendig wurde. Für pyrotechnische Artikel, die als offiziell erworben bezeichnet wurden, traf dies nur in 19 % der Fälle zu. In etwa einem Viertel der Fälle gab der Betroffene an, das auslösende Produkt nicht zuordnen zu können (Abb. 5). Das wirkt plausibel, da über alle Jahre mehr als die Hälfte der Patienten angibt, als Zuschauer oder Passant verletzt worden zu sein, und damit Angaben zum auslösenden Feuerwerksprodukt oft nicht möglich sind (Abb. 6).

### Unter den Versammlungsbeschränkungen weniger verletzte Zuschauer und Passanten

Der Anteil der verletzten Zuschauer oder in einer unklaren Situation Verletzten lag in den Jahren vor der Pandemie bei rund 62 %. Dieser Wert sank in den „Pandemiejahren“, als neben dem Verkaufsverbot Versammlungsbeschränkungen galten, auf 47 %. Da sich der Feuerwerkskörper während des Zündvorgangs näher am Körper befindet, könnte dies auch die etwas höhere Rate an Eigenverletzungen sowie an schweren Verletzungen und Begleitverletzungen in den Pandemiejahren erklären.

## Diskussion

### Vergleich von Anzahl und Schwere der Verletzungen über 6 Jahre, darunter 2 Jahre unter Pandemiebedingungen

Mit der von der DOG unterstützten Silvester-Umfrage, an der zuletzt 86 % der deutschen Notdienst leistenden Augenkliniken teilnahmen, können wir nach 6 Jahren Daten zu 2151 Patienten mit Augenverletzungen durch Feuerwerk analysieren. Durch das kontinuierliche und standardisierte Design unserer Untersuchung besteht die Möglichkeit, Daten aus 4 Jahren unter den bis zum Eintritt der Pandemie geltenden gesetzlichen Regelungen [1] mit den Ergebnissen aus den beiden Pandemiejahren 2020/21 und 2021/2022 zu vergleichen, in denen zur Entlastung der Krankenhäuser neben Beschränkungen von Versammlungen ein Verkaufsverbot für Feuerwerkskörper gesetzlich vorgegeben war. Damit tragen alle Teilnehmenden dazu bei, die Diskussion um das Verbot von privatem Feuerwerk oder alternativen Modellen eines Feuerwerks auf eine datenbasierte Grundlage zu stellen.

Wir wollen hier untersuchen, ob es, wie verschiedentlich als Gegenargument angeführt, durch das Verkaufsverbot zu einer Verlagerung auf nicht offizielle pyrotechnische Artikel mit vermehrt schweren Verletzungen gekommen ist.

Außerdem wollen wir unsere Ergebnisse nutzen, um die pandemiebedingten Regelungen in ihrem Stellenwert als Präventionsmaßnahme zu bewerten.

Im Vergleich der Gesamtzahlen von feuerwerksassoziierten Verletzungsfällen über die Jahre wird klar, dass das Verbot von privatem Feuerwerk in den Pandemiejahren 2020 bis 2022 zu einer deutlichen Abnahme der Verletzungsfälle geführt hat. Limitiert durch die geringen Fallzahlen ist der Anstieg von Verletzungsfällen im zweiten Pandemiejahr im Vergleich zum ersten nur als Tendenz zu bezeichnen, der jedoch mit schwereren Verletzungen durch nicht offizielle Pyrotechnik in Verbindung stehen könnte.

Unter den Pandemiebedingungen haben wir eine leicht erhöhte Zahl von Krankenhausaufnahmen und einen Anstieg der Verletzungen bei aktiven Feuerwerkern dokumentiert. Das könnte neben der etwas häufigeren Nutzung von nicht zugelassenen Feuerwerksartikeln auch ein Selektionseffekt der besonders risikofreudigen privaten Pyrotechniker sein oder aber auf die pandemiebedingten Versammlungsbeschränkungen zurückzuführen sein.

### Offiziell erworbene Feuerwerkskörper sind für die Mehrzahl der Augenverletzungen verantwortlich

Wie sehen in unseren Daten, dass Unfälle mit nicht offiziellen Feuerwerksartikeln zwar in fast der Hälfte der Fälle schwere, stationär zu versorgende Verletzungen nach sich ziehen. Es besteht ein statistisch signifikanter Unterschied im Anteil von nicht offiziellem Feuerwerk in Pandemiejahren zu Nicht-Pandemiejahren in Bezug auf den dadurch verursachten Schweregrad der Verletzungen.

**Figure 7:**
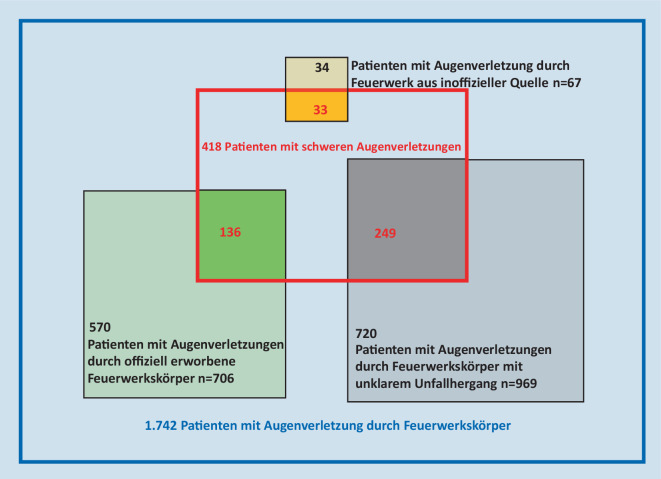

Die absolute Zahl aller Verletzungen durch nicht offizielle Feuerwerksartikel ist jedoch sehr viel geringer als die Zahl der Unfälle mit offiziell erworbenen Feuerwerksartikeln; 67 Unfälle mit nicht offizieller Pyrotechnik stehen 1675 Zwischenfällen mit offiziell erworbenen oder nicht benennbaren Feuerwerkskörpern gegenüber. Entsprechend sind 10-mal mehr Schwerverletzte aus Unfällen mit offiziellen oder nicht benennbaren Feuerwerkskörpern festzustellen (Abb. 7).

Unzweifelhaft sind auch Verletzungen aus der Gruppe mit unklarem Unfallhergang auf Feuerwerksartikel aus nicht offizieller Quelle zurückzuführen, aber das kann in den meisten Fällen und insbesondere dann nicht geklärt werden, wenn Unbeteiligte betroffen sind, was bei mehr als der Hälfte der Zwischenfälle zutrifft. Dieser hohe Anteil von 50–80 % Unbeteiligter ist vergleichbar mit den Ergebnissen nahezu aller Studien und stellt einen wichtigen Grund für die Forderung nach Einschränkung der privaten Feuerwerksaktivitäten dar [3].

Zusammenfassend ist ein Verkaufsverbot von privatem Feuerwerk eine effektive Maßnahme, um die Gesamtzahl der Verletzungen zu reduzieren. Die dadurch befürchtete Verwendung von nicht offiziellem Feuerwerk führt zwar relativ zu etwas mehr schweren Verletzungen, ist mit Blick auf die absoluten Zahlen aber überschaubar. Die Gesamtzahl und der Anteil schwerer Verletzungen durch den Gebrauch offiziell erworbener, also CE-zertifizierter pyrotechnischer Artikel überwiegt (Tab. 2).

Am Jahreswechsel 2020/21 verzeichneten wir 79 Augenverletzungen oder 1,1 bzw. 2,5 Patienten pro meldender Klink – der niedrigste Wert seit Erhebung.

Erstaunlich, dass sich im zweiten Jahr unter Pandemiebedingungen die Anzahl der Verletzungen im Vergleich zum Vorjahr auf 193 (2,5 Patienten je teilnehmender Klinik) mehr als verdoppelt hat, obwohl der Neuerwerb innerhalb Deutschlands nicht möglich war. Zu beobachten war am Jahreswechsel 2021/22 jedoch beim Erwerb von Pyrotechnikartikeln ein Ausweichen auf Nachbarländer. In Berlin etwa gibt es einen Shuttlebus zu einem Markt direkt hinter der polnischen Grenze, auf dem unter anderem Profifeuerwerk zu erwerben ist [4]. Im Dezember 2021 wurden größere Menge illegaler Feuerwerkskörper, die zum Weiterverkauf bestimmt waren, sichergestellt [5]. Aber auch Geschäfte in Tschechien und Belgien wurden von Deutschland aus angesteuert [6]. Importierte Produkte tragen jedoch zu einem Teil kein CE-Zertifikat und können höhere Sprengstoffmengen enthalten. Import und Nutzung sind ohne CE-Zertifikat nicht erlaubt [7]. Europaweite Regelungen wären wünschenswert.

### Öffentliches Feuerwerk ist sicherer als privates Feuerwerk

Professionelles Feuerwerk stellt im Gegensatz zu privatem Abbrennen eher keinen Risikofaktor dar: In den 6 Jahren unserer Umfrage fand sich lediglich eine Patientin, die während einer öffentlichen Feuerwerksshow als Zuschauerin leicht verletzt worden war.

Interessanterweise haben die pandemiebedingten Regelungen in den USA zu einer Zunahme der feuerwerksbedingten Verletzungen geführt. Begründet wird dies mit der Absage von öffentlichen Feuerwerksdarbietungen und dem stark angestiegenen Verkauf von Feuerwerkskörpern an Privatpersonen im Jahr 2020 [8] – auch dies ein Hinweis auf die Sicherheit von öffentlichem Feuerwerk.

### Alter der betroffenen Patienten

Das Spektrum der Patienten hat sich kaum geändert. Nach wie vor sind auch in unserer Auswertung männliche Patienten die dominierende Gruppe. Auch die Altersverteilung hat sich nach einer Rechtsverschiebung zum Jahreswechsel 2020/21, die vorrübergehend Anlass zur Hoffnung auf einen protektiven Effekt gegeben hat, im letzten Jahr wieder zu älteren Jugendlichen und jungen Erwachsenen zurückentwickelt. Unsere Daten sind bezüglich Alter und Geschlecht vergleichbar mit den Ergebnissen von Studien aus Leipzig [9], Halle [10] und Berlin [11], der Schweiz [12], den Niederlanden [13] und dem asiatisch-pazifischen Raum [3].

Wir stellen fest, dass in den vergangenen 6 Jahren 2151 Personen mit Augenverletzungen durch Feuerwerks- und Knallkörper in deutschen Augenkliniken dokumentiert worden sind; 424 von ihnen mussten stationär behandelt werden, und bei etwa der Hälfte dieser Patienten ist mit einer dauerhaften Sehverschlechterung oder Langzeitfolgen wie Narbenbildung an den Lidern oder der Hornhaut, Sekundärglaukomen oder Netzhautdefekten zu rechnen. Kinder und Jugendliche stellen rund ein Drittel aller Verletzten dar und sind unter den Betroffenen deutlich überrepräsentiert. Neben den direkten Folgen sind hier das Risiko von Amblyopieentwicklung und Spätfolgen wie Vernarbungen, PVR-Ablationes und Sekundärglaukomen [9, 10, 13] zu befürchten wie psychische Störung bei Kindern und Eltern [14].

### Datenerfassung in anderen Bereichen

Augenverletzungen stellen nach einer US-amerikanischen Studie nur etwa 18–30 % aller Verletzungen durch privat gezündetes Feuerwerk dar [15]. Um das gesamte Verletzungspotenzial von privater Pyrotechnik zu erfassen, sind Erhebungen aus anderen Fachrichtungen angestoßen worden. Eine Studie der HNO (Flockerzie, persönliche Kommunikation) publizierte im Oktober 2022 die ersten Ergebnisse. Eine deutschlandweite Datenerfassung der häufig involvierten Handchirurgie [16–18] ist wünschenswert.

### Ausblick

Einer Umfrage der Plattform „yougov“ zufolge, die die Deutsche Presseagentur in Auftrag gegeben hatte, unterstützten zwei Drittel aller Deutschen das von der Bundesregierung angeordnete Verkaufsverbot für pyrotechnische Artikel [19] und befürworten eine ähnliche Regelung auch ohne Pandemie [20, 21].

Mit unseren Ergebnissen wollen wir eine fundierte Datengrundlage für die gesellschaftliche Diskussion um private Feuerwerke in Deutschland schaffen, die dann politisch umgesetzt werden muss. Ein Konzept könnte sein, privates Feuerwerk durch kommunales zu ersetzen, das ausgebildete Feuerwerker z. B. aus den Reihen der kommunalen Feuerwehr zünden. Gemeinsam erlebtes, sicheres Feuerwerk höherer Qualität mit hochwertigen Herstellungsstandards, die etwa Kinderarbeit ausschließen, auch in Kombination mit umweltfreundlichen Produkten oder Lasershows kann zu einer deutlichen Reduktion von Augenverletzungen führen.

## Fazit für die Praxis

- Die pandemiebedingten gesetzlichen Regelungen einschließlich des Verkaufsverbots für Feuerwerksartikel und das umsichtige Verhalten der Bevölkerung haben in den vergangenen beiden Jahren zu einem deutlichen Rückgang der feuerwerksassoziierten Augenverletzungen geführt. Die Nutzung als inoffiziell erworben bezeichneter Feuerwerkskörper hat etwas zugenommen, dennoch ist in den vergangenen Jahren der größte Teil der dokumentierten Augenverletzungen durch als offiziell erworben bezeichnete, also CE-zertifizierte Feuerwerkskörper verursacht worden.
- Öffentliche Feuerwerke sind sicherer und als Alternative zu empfehlen.

## Supplementary Information




