# Supplementary figures and images for: Pandemic-related sales ban of fireworks in Germany leads to a significant reduction of firework-related eye injuries
Source: Ophthalmologie. 2022 Nov 30;119(12):1257–66. [Article in German] doi: 10.1007/s00347-022-01778-1 (PMC9713168; doi:10.1007/s00347-022-01778-1)

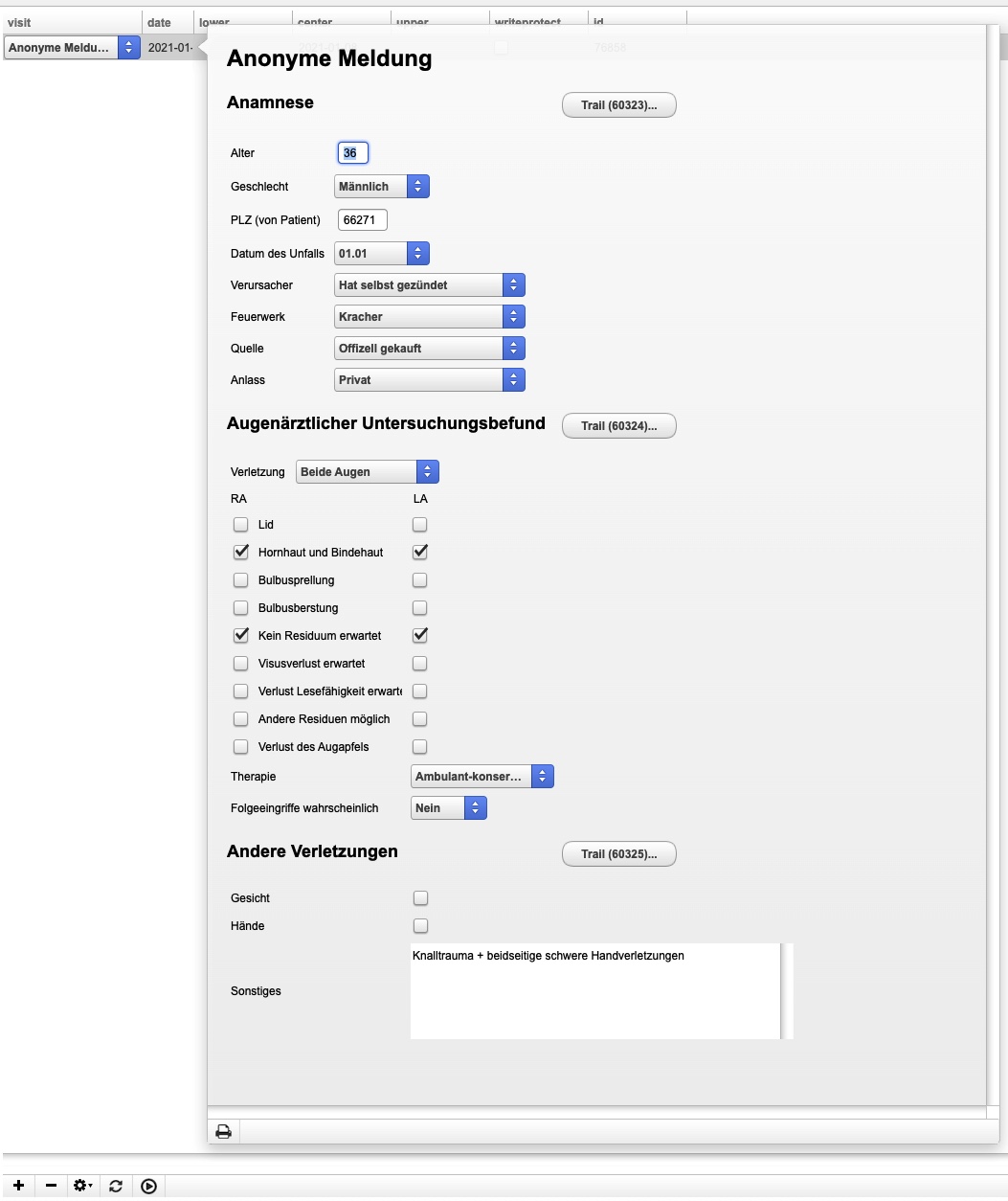

Supplement: Supplementary file 1 [file 347_2022_1778_MOESM1_ESM.jpg]
